# Supplementary figures and images for: Validation of N Protein Antibodies to Diagnose Previous SARS-CoV-2 Infection in a Large Cohort of Healthcare Workers: Use of Roche Elecsys® Immunoassay in the S Protein Vaccination Era
Source: Viruses. 2023 Apr 7;15(4):930. doi: 10.3390/v15040930 (PMC10146079; doi:10.3390/v15040930)

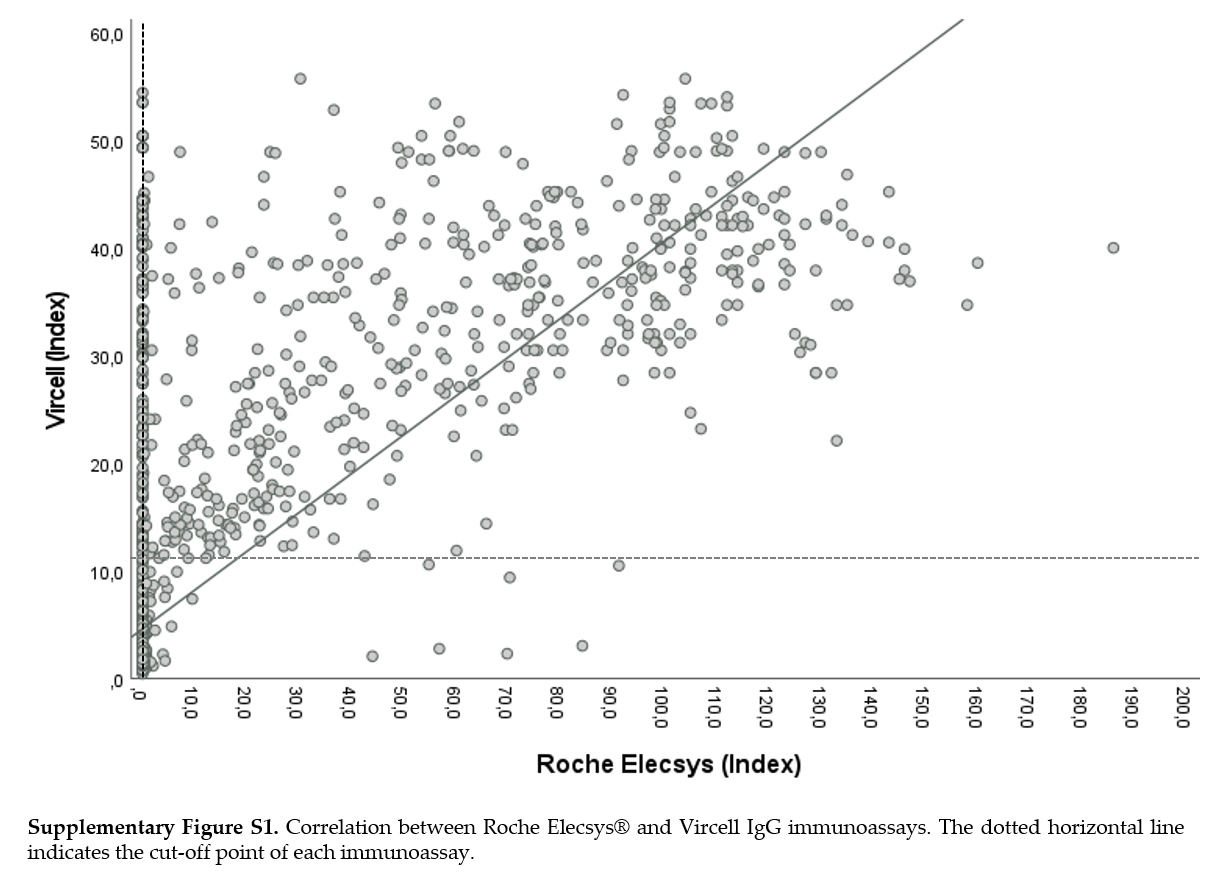

Supplement: Supplementary file 1 [file viruses-15-00930-s001.zip › viruses-2223042-figures.png]
